# Medical Opinion and Movement

**Published:** 1907-07-06

**Authors:** 


### Medical Opinion and Movement.

It is one thing to get justification from the law, but quite another to obtain pecuniary compensation. This is evidenced in the case of Dr. Bagley at Manchester. Dr. Bagley was charged with obstructing and assaulting the police in the execution of their duty. He was called in to attend an old man of feeble intellect, who had attempted suicide by cutting his throat. The police insisted on taking him to the Ancoats Hospital, in spite of Dr. Bagley's statement that he was unfit for removal and could be tended and guarded at home. The magistrate decided that the police had no right to remove the man without a warrant, and the Chief Constable issued instructions that under such circumstances the police must act in accordance with the doctor's instructions. Although Dr. Bagley was in this way fully justified in his action, he incurred legal expenses to the extent of about £30 over the affair, and he applied to the Watch Committee for his costs. These have been refused, in spite of further representations on his behalf by the local Council of the British Medical Association. Apart from the magistrate's decision, the order of the Chief Constable is such a complete confession of error on the part of the police, that one would suppose some compensation would have been readily granted to the doctor to meet expenses incurred in carrying out his professional duties.

It is often difficult to obtain definite evidence of alcoholic excess in cases of failing heart, in which alcohol may be suspected as the cause on clinical grounds. Any clinical sign therefore which will aid the physician to draw his own independent conclusions is of considerable utility. Dr. R. T. Williamson, of Manchester, is of opinion that the loss of the tendo Achillis reflex affords such a sign. Of 21 cases of alcoholic heart failure he has found this reflex absent in 18 cases. He regards this sign as evidence of slight incipient neuritis, and points out that it is one of the first reflexes to disappear in such conditions. Only seven cases out of the 21 had absent knee-jerks, and these were among those with advanced symptoms of failing heart. In 100 cases of cardiac disease not due to alcoholism, only two showed absent tendo Achillis jerks, and in these the knee-jerks were also absent, and there was the Argyll-Robertson pupil. In considering the value of the absence of the tendo Achillis reflex in relation to alcoholic excess, other signs indicative of nerve lesions must of course be taken into account. Thus in both these cases there was aortic valvular disease, and the absent reflexes and Argyll-Robertson pupils were due to incipient tabes dorsalis.

The first part of the report on vital statistics for the decennial period, 1891-1900, prepared by Dr. Tatham, superintendent of statistics, and issued from the office of the Registrar-General for England and Wales, has just made its appearance. This is the fifth volume in succession, and therefore allows of a retrospect over a period of half a century. In such a retrospect the essential points of general interest are the comparative rates of births and deaths in the successive periods. As pointed out by Dr. Tatham in his introductory letter, these figures must be considered together. Thus the apparent fall in the general death-rate is from 22.28 per 1,000 living in 1841-50 to 18.19 per 1,000 in 1891-1900. These figures do not, however, represent the actual diminution in death-rate. During this time the birth-rate has also fallen considerably, and such a fall materially modifies the age constitution of the population, and as the mortality-rate varies for the different ages, a corresponding change in the general death-rate results. According to Dr. Tatham's calculation with the same age constitution in 1841-50, as ruled in 1891-1900, the death-rate during the earlier period would have been 21.74 per 1,000, so that the actual fall in mortality is from 21.74 per 1,000 to 18.19 per 1,000. There has unfortunately been no such corresponding fall in infant mortality. Thus in 1891-1900 the death-rate at ages under one year was 181.2, which is the same figure as that for the period 1861—70. Comparison between the death-rates in country and urban districts show that it is the conditions of town life which are especially responsible for this maintenance of a high infant mortality.

An important experiment has been carried out by Dr. W. Fletcher in the Kuala Lumpur Lunatic Asylum in the Malay States to test the supposed causal relation between the incidence of the disease beri-beri and the use of uncured rice as a food. An epidemic of the disease broke out in the asylum in 1905, and out of 219 lunatics 94 were affected during the year, with a mortality of 27. Uncured rice formed the chief constituent of diet for the inmates. At the end of the year it was decided to place half the lunatics on cured rice—that is, rice that is stored after being boiled, and then husked. This system of feeding was continued throughout last year, and during that time out of 120 inmates fed on uncured rice 34 suffered from beri-beri, and 18 died; whereas among 123 patients fed on cured rice there were only two cases of beri-beri, both of whom were suffering from the disease on admission, and no deaths. The two batches of patients were kept in separate wards and fed at different times, and separate cooking and feeding utensils were used, but otherwise the patients were allowed to associate together, and at the half-year the two batches were changed over to each other's apartments. Ten of the patients living on uncured rice and suffering from beri-beri speedily recovered when transferred to the other side, and on the other hand, of four patients hitherto fed on cured rice, when changed over to the uncured rice, two developed the disease, and one died. These facts are not only important evidence in support of the supposition that uncured rice is in some way responsible for the occurrence of beri-beri, but they also go to disprove the theory of the disease being microbic and infective in character. What the causal relation actually is remains as yet unsolved; whether there is some poison in the rice, or some nutritive deficiency rendering the individual susceptible to the disease.

